# Study of the causal relationship between gastroesophageal reflux disease and hypertension through two-sample Mendelian randomization analysis

**DOI:** 10.3389/fcvm.2024.1326348

**Published:** 2024-09-24

**Authors:** Weige Li, Qian Wang, Wenjie Li, Xiang Liu, Zuobin Li, Qi Dai

**Affiliations:** ^1^ Graduate School, Jiangxi University of Chinese Medicine, Nanchang, Jiangxi, China; ^2^ Department of Gastroenterology, The Affiliated Hospital of Jiangxi University of Chinese Medicine, Nanchang, Jiangxi, China

**Keywords:** causal inference, gastroesophageal reflux disease, hypertension, Mendelian randomization, gastroenterology

## Abstract

**Objective:**

The purpose of this study was to investigate the causal relationship between gastroesophageal reflux disease (GERD) and hypertension using a two-sample Mendelian randomization analysis.

**Methods:**

The associated data of GERD with hypertension were derived from the genome-wide association study (GWAS) database, and two-sample Mendelian randomization (MR) analysis was performed using methods including inverse variance weighting (IVW), MR-Egger, and weighted median (WM) to investigate the causal association between GERD and hypertension.

**Results:**

A total of 16 single nucleotide polymorphisms (SNPs) strongly associated with GERD were screened out, and the IVW suggested a causal relationship between GERD and hypertension (OR: 1.057; 95% CI: 1.044–1.071; *P* < 0.05). The weighted median also showed a similar relationship (OR: 1.051, 95% CI: 1.032–1.07; *P* < 0.05). In addition, no heterogeneity or horizontal pleiotropy was observed, suggesting a robustness of the outcome.

**Conclusion:**

There is a positive causal relationship between GERD and hypertension.

## 1 Introduction

Hypertension is an important comorbidity and risk factor for cardiovascular disease (CVD) and is one of the leading causes of death due to CVD (1–3). It has been reported that about one-third of adults worldwide suffer from hypertension, and the prevalence is increasing year by year (4–6). The prevention and treatment of hypertension have become a major public health problem to be solved. Even though patients actively cope with it through improvements in lifestyle and medication, the percentage of hypertension treated and controlled satisfactorily remains low (7). This phenomenon arises from the intricate nature of the mechanisms governing blood pressure regulation, and a comprehensive comprehension of the etiology of hypertension remains elusive within the current body of knowledge.

Gastroesophageal reflux disease (GERD) is a common gastrointestinal disease that is caused by the reflux of gastric contents into the esophagus and mainly manifests as heartburn and reflux (8, 9). Besides, GERD is clinically correlated with many symptoms and diseases outside of the GI (gastrointestinal) tract in addition to the common GI symptoms (10, 11). A study has shown that GERD is closely related to the progression of hypertension and may be one of the important risk factors (12). Li et al. (13) found that the incidence of hypertension in GERD patients was significantly higher than that in non-GERD patients. After omeprazole antacid treatment, the incidence of hypertension in patients was significantly reduced, suggesting that PPI drugs could help hypertensive patients return to normal blood pressure. In addition, the study showed that antacids significantly reduced the blood pressure in patients with essential hypertension, suggesting that the treatment of GERD may help normalize the blood pressure in hypertensive patients. However, another study revealed no correlation between hypertension and GERD (14). Therefore, the relationship between GERD and hypertension remains controversial.

For the first time, the Mendelian randomization (MR) method was used to investigate whether there is a causal relationship between GERD and hypertension. MR is a statistical method based on whole-genome sequencing data, which can effectively reduce bias and is used to expose causal relationships (15). Compared with estimates from observational studies, MR estimates are less susceptible to potential reverse causality and confusion, as genetic variants are randomly distributed at conception and largely independent of environmental and life factors (16).

## 2 Research methods and materials

### 2.1 Study design

To investigate the causal relationship between GERD and hypertension, firstly, we derived the associated data of GERD with hypertension from the publicly available GWAS database, screened out qualified single nucleotide polymorphisms (SNPs), and determined the causal association between GERD and the risk of onset of hypertension by using various statistical methods.

### 2.2 Data sources

The GERD-related data were obtained from the IEU (Integrative Epidemiology Unit) open GWAS database at https://gwas.mrcieu.ac.uk/datasets/ebi-a-GCST90000514/. The sample size of this dataset was 602,604, which included 129,080 patients with GERD and 473,524 control cases, all of European origin, and the number of SNPs was 2,320,781. Data on hypertension were obtained from the IEU open GWAS database at https://gwas.mrcieu.ac.uk/datasets/ukb-b-12493/. The database has a sample size of 463,010, including 54,358 hypertensive patients and 408,652 control cases, all of European origin, and the number of SNPs was 9,851,867.

### 2.3 Selection of instrument variables

**Figure 1:**
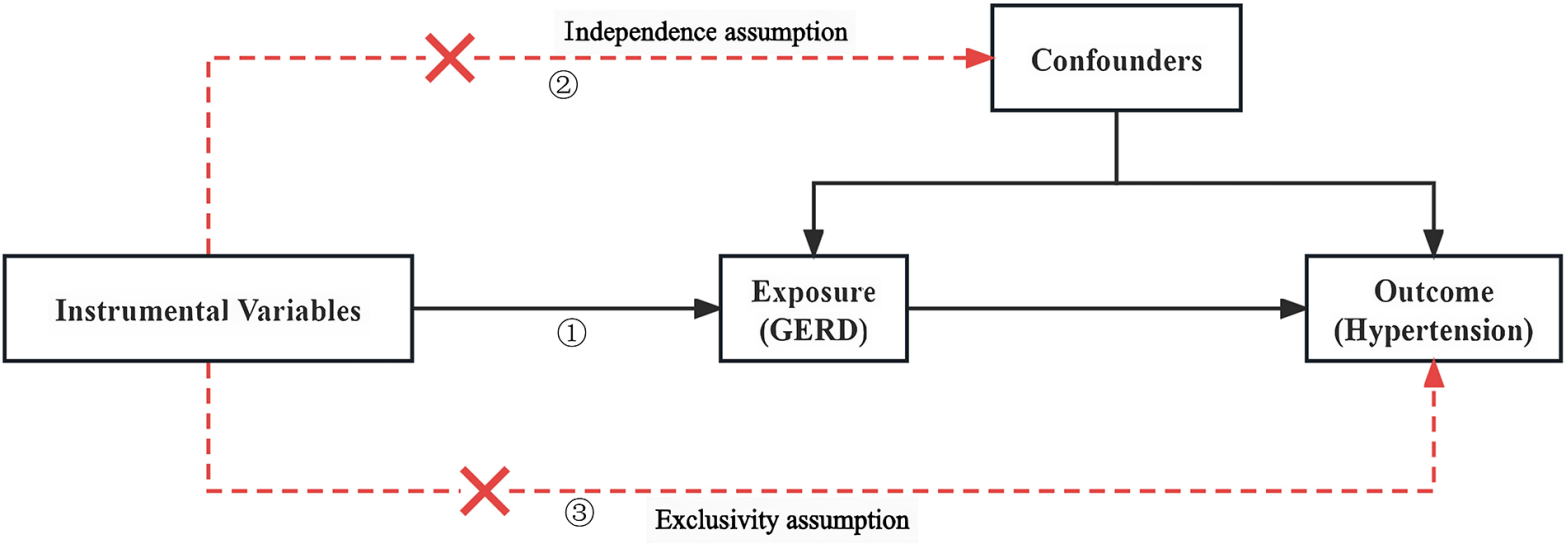
Two-sample Mendelian diagrammatic model. **(1)** Correlation hypothesis: SNPs are strongly associated with exposure; **(2)** Independence hypothesis: SNPs are not associated with confounders; **(3)** Exclusivity hypothesis: SNPs are not associated with outcomes.

The following three assumptions for the two-sample Mendelian randomization (17) analysis were fulfilled: (1) The correlation assumption: the SNPs were strongly associated with exposure; (2) The independence assumption: the SNPs were not associated with confounders; and (3) The exclusivity assumption: the SNPs were not associated with the outcome see Figure 1. We screened out SNPs that were strongly associated with GERD (*P* < 5 × 10^−8^) from the database, ensuring that the correlation assumption was met. Additionally, we excluded SNPs with chain disequilibrium to ensure an *r*^2^ value less than 0.001 and a genetic distance greater than 10,000 kb, while maintaining the independence among SNPs (18). We used the PhenoScanner database (www.phenoscanner.medschl.cam.ac.uk) to identify and exclude SNPs associated with the confounders, ensuring that the independence assumption was met. We also calculated the *F*-statistics to assess the presence of a weak instrument bias. To further validate the association assumption, we used the computational formula F = [(*N*-*K*-1)/*K*] × [*R*^2^/(1-*R*^2^)], where *N* refers to the sample size of the exposure factors, *K* refers to the number of instrument variables, and *R*^2^ represents the variation percentage of the exposure factors explained by the instrument variables. We selected instrument variables with F greater than 10 (19), during the analysis, SNPS with palindromic structure were automatically excluded, and instrumental variables significantly correlated with GERD were finally obtained.

### 2.4 Statistical processing

To estimate the causal effect between GERD and hypertension, we employed the methods of inverse variance weighted (IVW), MR-Egger, and weighted median (WM), with IVW being the predominant method (20). In addition, we assessed the heterogeneity of instrument variables using Cochran's *Q*-test, *P* > 0.05 indicates no heterogeneity. To determine the presence of outliers and horizontal pleiotropy, we conducted the MR-PRESSO (Mendelian randomization pleiotropy residual sum and outlier) test. Identify any factors that may lead to horizontal pleiotropy using the MR-PRESSO method, and if outliers are found, delete them and perform MR analysis again. The intercept term of MR-Egger regression was employed to test for potential horizontal pleiotropy of SNPs (21). *P* < 0.05 indicates the existence of horizontal pleiotropy, indicating that instrumental variables can affect outcomes through factors other than exposure, which violates the independence assumption of the three major hypotheses. Lastly, we used the leave-one-out method to evaluate the magnitude of the impact of individual SNPs on the results of causal association estimation, thereby further validating the robustness of the findings (22).

R 4.2.2 software, TwoSampleMR software package, and MRPRESSO software package were used for statistical processing. When *P* < 0.05, the value was considered statistically significant.

### 2.5 Ethics

For this study, we used data obtained from publicly available databases and there were no ethical issues.

## 3 Results

### 3.1 Final instrument variables included in MR analysis

We obtained 16 SNPs, and all the F-values included in the MR analysis were greater than 10, indicating that there was no weak instrument bias and that the outcomes were reliable, as shown in Table 1.

**Table 1:** A total of 16 SNPs.

SNP	CHR	EA	OA	GERD			Hypertension		
				β	SE	*P*	β	SE	*P*
rs10010963	4	T	C	−0.0269803	0.00494665	0.000000049	−0.000319058	0.000685687	0.640000000
rs11953061	5	T	C	0.0281599	0.00508694	0.000000031	0.00235072	0.000706228	0.000870001
rs12204714	6	T	C	−0.028817	0.00499422	0.000000008	−0.00140808	0.000691022	0.042000100
rs12453010	17	T	C	0.0296967	0.00493315	0.000000002	0.00150441	0.000684597	0.028000100
rs1479405	12	T	C	0.0314843	0.0051514	0.000000001	0.00195284	0.000713078	0.006199980
rs1883842	20	G	T	0.0308332	0.00536826	0.000000009	0.00295892	0.000745325	0.000072000
rs2106353	7	T	G	0.0367491	0.00572505	0.000000000	0.0022964	0.000793934	0.003799970
rs3828917	6	T	G	0.0671113	0.0120054	0.000000023	0.00228413	0.00166456	0.170000000
rs3863241	8	T	C	0.0324982	0.00481521	0.000000000	0.000966346	0.000666742	0.150000000
rs4300861	2	T	C	0.0307132	0.00494886	0.000000001	0.00318525	0.000686598	0.000003500
rs569356	1	G	A	−0.037919	0.00690971	0.000000041	−0.000797499	0.000960207	0.410000000
rs7527682	1	G	A	−0.026684	0.00482174	0.000000031	−0.00197335	0.000667666	0.003099990
rs7600261	2	T	C	0.0338034	0.00522051	0.000000000	0.00119707	0.000723307	0.098000900
rs761777	10	G	A	0.0345341	0.00554469	0.000000000	0.00150577	0.000763573	0.049000400
rs9396740	6	A	G	−0.031493	0.0055587	0.000000015	−0.0023147	0.00076796	0.002599980
rs9529055	13	A	G	0.0266604	0.00481637	0.000000031	0.00176907	0.000666722	0.008000000

CHR, chromosome; EA, effector allele; OA, non-effector allele; β, allelic effect value; SE, standard error of β.

### 3.2 MR results

**Figure 2:**
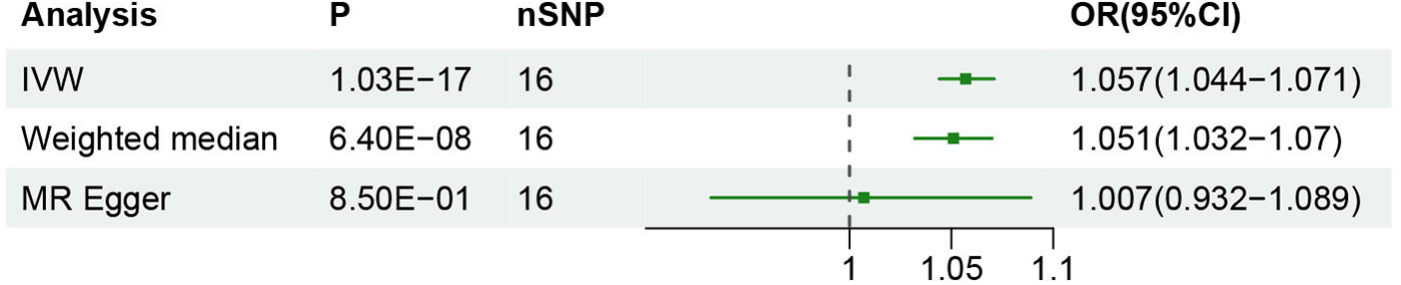
Results of two-sample Mendelian randomization analysis.

The inverse variance weighted (IVW) method estimated an odds ratio (*OR*) of 1.057 [95% confidence interval (95% CI): 1.044–1.071; *P* < 0.05], indicating a causal relationship between GERD and hypertension. Similarly, the weighted median method showed an *OR* of 1.051 [95% CI: 1.032–1.07; *P* < 0.05] supporting a comparable relationship. Furthermore, the MR Egger regression analysis indicated that the final results were not affected (OR: 1.007, 95% CI: 0.932–1.089; *P* > 0.05) see Figure 2 for details. The results demonstrated a strong relationship between GERD and the occurrence of hypertension.

### 3.3 Sensitivity analysis

**Figure 3:**
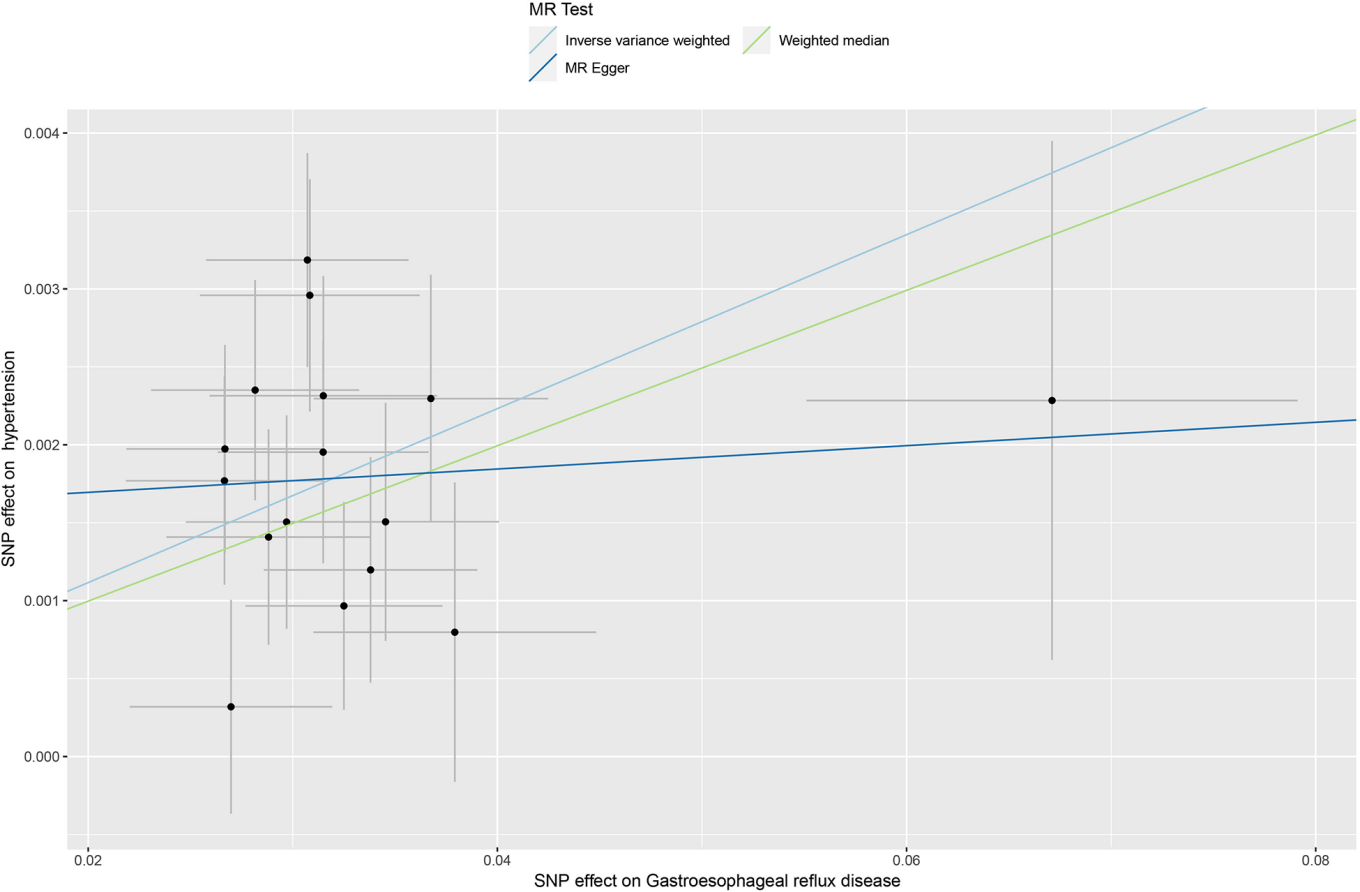
Scatterplot of two-sample Mendelian randomization.

**Figure 4:**
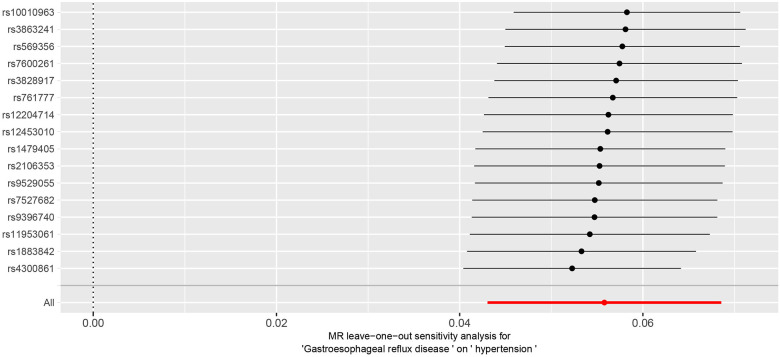
“Leave-one-out” forest plot.

The Cochran *Q*-test results yielded a *P*-value of 0.2, indicating the absence of heterogeneity. The MR-Egger intercept term was close to 0 (*P* = 0.2), indicating that the results of this study were minimally affected by horizontal pleiotropy and therefore robust see Figure 3 for details. Additionally, MR PRESSO suggested the absence of outliers and horizontal pleiotropy (*P* = 0.4). Finally, the IVW method was tested using the leave-one-out method, and no single SNP significantly affected the robustness of the results, further confirming the reliability of the final results see Figure 4.

## 4 Discussion

GERD is a prevalent epidemic that has reached alarming levels of 10%–20% in Western countries (23). In the East Asian region, which historically had the lowest rate worldwide, the prevalence of GERD has also been increasing over time (24). Meanwhile, a study revealed that the number of adults with hypertension has increased from 648 million in 1990 to 1,278 million in 2019, indicating an upward trend (25). Consequently, the prevention and treatment of GERD and hypertension have been increasingly emphasized. A growing body of literature has also revealed a possible direct or indirect relationship between the two. By retrospectively analyzing a GERD database of 1,052 patients who had undergone laparoscopic fundoplication (LF), Hu et al. (26) found that a large proportion of patients with GERD combined with hypertension were stabilized in the normal range postoperatively after treatment with LF. The control of hypertension on average was significantly lower in all patients included in the study, which suggests that hypertension may be secondary to gastroesophageal reflux in some patients. Another study (27) found that rabeprazole can increase human plasma nitric oxide level, and nitric oxide has the function of vasodilating, anti-inflammatory, and inhibiting smooth muscle cell growth, and can effectively improve blood pressure level, especially for patients with gastroesophageal reflux disease accompanied by hypertension. GERD is also an important risk factor for cardiovascular diseases (CVD), and it has been reported in the literature that among patients with GERD, the incidence of cardiovascular diseases such as coronary artery disease (CAD), atrial fibrillation (AF), and acute myocardial infarction (AMI) is significantly higher than that in patients without GERD (28–30). In this study, we found a positive causal relationship between GERD and hypertension using the Mendelian randomization method. This finding suggests that GERD is an important risk factor for hypertension, which has significant implications for the prevention, diagnosis, and treatment of hypertension.

The specific mechanism by which GERD increases the risk of hypertension is currently unknown, and the available research suggests the following possible explanations. Since the activity of the digestive tract is closely related to the autonomic nerves, sympathetic excitation caused by esophageal stimulation during GERD episodes may be one of the explanations for GERD-induced hypertension (31–33). There is another theory that has been proposed to explain the relationship between hypertension and GERD. The theory suggests that the increased secretion of gastric acid during the onset of GERD in patients with GERD stimulates the activation of caudal solitary complex (cSC) neurons. The cSC neurons exhibit dual functionality, encompassing digestive reflexes and the modulation of cardiac and pulmonary functions. These functions manifest in gastrointestinal symptoms characterized by a reduction in the tone of the lower esophageal sphincter (LES), resulting in the reflux of gastric contents. Additionally, cSC neurons influence cardiac and pulmonary regulation by precipitating an elevation in the proportion of carbonic acid within the bloodstream, thereby inducing an increase in blood pressure via the mechanism of hypercapnia (34). Through studies on animal models and molecular data, it has been found that many inflammatory mediators such as IL-8, IL-6, and IL-1β, among others, exist in the pathogenesis of GERD. The main mechanism of esophageal injury caused by GERD may be the cytokine-mediated immune response, and these inflammatory mediators have been continuously discovered and studied in recent years (35). Meanwhile, as researchers have gained a deeper understanding of inflammatory mediators, numerous studies have demonstrated that inflammatory responses are also involved in the progression of hypertension (36). Thus, long-term chronic inflammation may contribute to the progression of hypertension by producing inflammatory mediators and participating in angiogenesis, while both innate and adaptive immunity can contribute to elevated blood pressure by triggering vascular inflammation and microvascular remodeling (37). Therefore, the involvement of GERD in the progression of hypertension through inflammatory cytokines is also one of the possible mechanisms by which GERD causes hypertension.

There may be mechanisms other than those mentioned above for GERD-induced hypertension. Only a limited number of studies have investigated the potential link between GERD and hypertension, and existing studies on the association between GERD and hypertension are mostly observational and lack in-depth analysis at the genetic level. Consequently, it is imperative to delve deeper into the underlying mechanisms responsible for GERD-induced hypertension, to elucidate the intricate signaling pathways associated with this phenomenon. Such an investigation holds the potential to offer valuable insights into the etiological framework of hypertension and may unveil prospective therapeutic targets for clinical intervention. This study provides theoretical support for this endeavor. The bidirectional feedback between GERD and hypertension can be investigated in subsequent studies to explore whether there is a causal relationship between hypertension and the development of GERD, thus further elucidating the pathogenic relation between GERD and hypertension.

This study has some limitations: First, the samples included in the study were of European origin, and it cannot be proved that the same results can be obtained in other regions and ethnic groups; second, since the data used are not original, we can only estimate the approximate causal relationship between the two, not determine their specific causal link; third, this study only produced statistical results and did not further investigate the mechanism by which GERD causes hypertension.

## 5 Conclusion

In summary, the two-sample MR method was used in this study for the first time to infer the causal relationship between GERD and the development of hypertension and produced a preliminary conclusion that a positive causal relationship exists between the two. Although the specific mechanism was not elucidated, this study provides another perspective on the risk factors for the development of hypertension. Timely diagnosis and treatment of GERD can significantly improve and reduce the development of associated hypertension and deserves further research and application.

## Data Availability

The original contributions presented in the study are included in the article/Supplementary Material, further inquiries can be directed to the corresponding author.
